# Discovery of a small-molecule inhibitor of the TRIP8b–HCN interaction with efficacy in neurons

**DOI:** 10.1016/j.jbc.2022.102069

**Published:** 2022-05-24

**Authors:** Ye Han, Iredia D. Iyamu, Matthew R. Clutter, Rama K. Mishra, Kyle A. Lyman, Chengwen Zhou, Ioannis Michailidis, Maya Y. Xia, Horrick Sharma, Chi-Hao Luan, Gary E. Schiltz, Dane M. Chetkovich

**Affiliations:** 1 Department of Neurology, Vanderbilt University Medical Center, Nashville, Tennessee, USA; 2 Center for Molecular Innovation and Drug Discovery, Northwestern University, Evanston, Illinois, USA; 3 High Throughput Analysis Laboratory and Department of Molecular Biosciences, Northwestern University, Evanston, Illinois, USA; 4 Department of Biochemistry and Molecular Genetics, Northwestern University, Chicago, Illinois, USA; 5 Department of Neurology, Stanford University, Palo Alto, California, USA; 6 Robert H. Lurie Comprehensive Cancer Center, Northwestern University Feinberg School of Medicine, Chicago, Illinois, USA; 7 Department of Pharmacology, Northwestern University, Chicago, Illinois, USA; 8 Department of Chemistry, Northwestern University, Evanston, Illinois, USA

**Keywords:** major depressive disorder, small molecule, TRIP8b, HCN, hippocampus, h current, I_h_, DCM, dichloromethane, EA, ethyl acetate, eGFP, enhanced GFP, ESI, electrospray ionization, FA, formic acid, HA, hemagglutinin, HCN, hyperpolarization-activated cyclic nucleotide–gated, MDD, major depressive disorder, STD, saturation transfer difference

## Abstract

Major depressive disorder is a critical public health problem with a lifetime prevalence of nearly 17% in the United States. One potential therapeutic target is the interaction between hyperpolarization-activated cyclic nucleotide–gated (HCN) channels and an auxiliary subunit of the channel named tetratricopeptide repeat–containing Rab8b-interacting protein (TRIP8b). HCN channels regulate neuronal excitability in the mammalian hippocampus, and recent work has established that antagonizing HCN function rescues cognitive impairment caused by chronic stress. Here, we utilize a high-throughput virtual screen to find small molecules capable of disrupting the TRIP8b–HCN interaction. We found that the hit compound NUCC-0200590 disrupts the TRIP8b–HCN interaction *in vitro* and *in vivo*. These results provide a compelling strategy for developing new small molecules capable of disrupting the TRIP8b–HCN interaction.

Major depressive disorder (MDD) is a critical public health problem with many patients failing to respond to existing pharmacotherapies. Most antidepressants focus on monoaminergic neurotransmitters, but these drugs take several weeks to achieve their therapeutic effect and fail to treat some of the most debilitating aspects of MDD, including cognitive impairment (1, 2). As such, there is a need for new and synergistic therapies. Cognitive impairment in MDD is often localized to the hippocampus, a key limbic structure that plays an essential role in both affect and memory. While it remains unclear how precisely stress impairs hippocampal function, many mechanisms have been described and a common endpoint is a loss of hippocampal excitability (3). For example, chronic stress leads to a reduction in the strength of glutamatergic inputs to CA1 pyramidal neurons while monoaminergic antidepressants rescue this activity (4, 5, 6, 7). In addition to changes in synaptic function, the intrinsic excitability of CA1 pyramidal neurons is also altered by chronic stress and limits hippocampal excitability (8). Hyperpolarization-activated cyclic nucleotide–gated (HCN) channels play an essential role in regulating CA1 pyramidal neuron excitability and animal models subjected to chronic stress led to higher levels of hippocampal HCN expression (8). There are four isoforms of these noninactivating voltage-sensitive channels (HCN1-4) and all are permeable to Na^+^ and K^+^ but differ in their sensitivity to second messengers (9). HCN1 and HCN2 are expressed at high levels in the mammalian hippocampus and are open at the resting membrane potential of CA1 pyramidal neurons where they limit cellular excitability by minimizing the change in membrane voltage in response to synaptic input (10, 11).

Multiple lines of evidence suggest that limiting hippocampal HCN channel function may be a relevant therapeutic target for the treatment of MDD (8, 12, 13, 14, 15). Knockdown (8, 13) or knockout (KO) (15) of hippocampal HCN channels has been associated with an increase in antidepressant-like behavior (behavior that is similar to that of an animal given an antidepressant such as fluoxetine (16)). Moreover, we recently reported an increase in HCN channel expression in the postmortem hippocampi of human patients with MDD, which is consistent with findings from animal models of chronic stress (8, 17, 18). Although, these results suggest that directly targeting HCN channels with a small molecule antagonist could be an effective antidepressant strategy, HCN channels are also expressed in cardiac tissue where they play an essential role in pacemaking (19, 20). As such, directly targeting HCN is likely to cause bradycardia similar to the blood–brain–barrier impermeable HCN channel antagonist ivabradine (20).

To avoid inhibiting cardiac HCN channels, our group has focused on a subunit of HCN named tetratricopeptide repeat–containing Rab8b-interacting protein (TRIP8b) (21, 22, 23). TRIP8b binds HCN pore-forming subunits in dorsal CA1 pyramidal neurons in a 1:1 ratio and regulates both HCN channels subcellular trafficking and opening probability (24, 25). These two roles of TRIP8b are performed by distinct domains of TRIP8b. The N terminus of TRIP8b is made up of variably spliced exons containing different functional domains that mediate protein–protein interactions between TRIP8b and proteins involved in subcellular transport. For example, inclusion of an adapter protein consensus sequences leads to clathrin-mediated endocytosis of the TRIP8b–HCN complex (25). Although the physiologic function of many of these splice isoforms remains opaque, nearly all of them are involved in increasing surface expression of HCN channels in neurons such that KO of TRIP8b leads to a dramatic reduction in hippocampal HCN channels (14). Genetic KO of TRIP8b also leads to an antidepressant-like phenotype similar to that of HCN KO animals (15). Moreover, viral rescue experiments have confirmed that the behavioral phenotype of TRIP8b KO mice is specifically linked to the function of TRIP8b in transporting HCN channels into the distal dendrites of CA1 pyramidal neurons (14).

Although initial reports suggested a complete lack of TRIP8b in cardiac tissue (19, 26), a more recent report suggests that TRIP8b is expressed at low levels in some intracardiac nervous tissue and may play a subtle role in setting the atrial refractory period (27). However, there was no difference in propensity to arrhythmias in hearts lacking TRIP8b and virtually all electrophysiological properties were unchanged (27). Combined, these studies suggest that targeting TRIP8b will not lead to unwanted cardiac side effects.

To validate the TRIP8b–HCN interaction as a therapeutic target, we recently employed a chemogenetic approach to disrupt the interaction in mice (17). Following chronic social defeat, an animal model of chronic stress, mice identified as “susceptible” to defeat based on a change in social behavior also develop impairments in cognition and motivated behavior. However, we demonstrated that chemogenetically disrupting the TRIP8b–HCN interaction in susceptible mice rescued cognitive function and improved motivated behavior (17). These results are consistent with the hypothesis that a small molecule designed to disrupt TRIP8b binding could be used to treat the impairments in cognition that accompany chronic stress.

TRIP8b binds to HCN channels at two distinct sites, although disrupting either site is sufficient to limit TRIP8b-mediated HCN dendritic trafficking. First, TRIP8b binds to the cytoplasmic cyclic nucleotide–binding domain of the channel. HCN channels are voltage-gated channels that open in response to hyperpolarization and mediate a nonspecific cationic current (I_h_) with a reversal potential of approximately −25 mV (28). However, binding of TRIP8b to the cytoplasmic cyclic nucleotide–binding domain of the channel hyperpolarizes the half-activation potential (V_50_, the membrane potential at which 50% of channels are open) so that more hyperpolarized voltages are required to maximally open the channel (21, 25, 29, 30). Note that this shift in opening probability occurs independently of the effect of TRIP8b on surface trafficking of HCN channels that is described previously (30). The second TRIP8b–HCN interaction site has previously been crystallized and occurs between the tetratricopeptide repeat domains of TRIP8b and the C terminus of HCN channels (24). Although protein–protein interactions are difficult targets for small molecule development, we reasoned that the deep pocket formed by the tetratricopeptide repeat domains of TRIP8b (24) would be amenable to small molecule disruption. We previously developed AlphaScreen and fluorescence polarization based high-throughput screening assays to characterize small molecules capable of disrupting the TRIP8b–HCN interaction (31). In addition, the availability of a high-quality crystal structure allowed for *in silico* screening to identify additional inhibitors. Here, we report the results of our *in silico* screen and subsequent *in vitro* and *in vivo* characterization of a resulting small molecule inhibitor using a variety of biochemical, cellular, and biophysical assays. These results provide evidence that small molecule inhibitors may be used as research tools to study the TRIP8b–HCN interaction and potentially serve as a starting point to develop a novel class of antidepressants.

## Results

### *In silico* high-throughput screening of TRIP8b

The ZINC database (32) was subjected to a panel of Pan-assay interference compound (PAINS) substructure filters with Smiles ARbitrary Target Specifications (SMARTS) strings to eliminate promiscuous and nondrug-like molecules that interfere with the functionality of the target proteins (33). Filtering resulted in a set of approximately 11.2 million commercially available compounds for further screening. The curated dataset was then subjected to the LigPrep module of Schrödinger in OPLS3 force field at pH 7.4 ± 1 retaining the specific chirality to produce a low energetic 3D structure for each molecule.

The protein preparation engine implemented in the Schrödinger software suite was utilized to prepare the TRIP8b protein for small molecule–docking simulations. Analysis of the atomic structure of TRIP8b (Protein Data Bank code 4EQF), which is complexed with an HCN2 carboxy-terminus peptide, showed that the critical interactions between the peptide and TRIP8b included the E67, N99, R236, N205, R206, N213, and N240 residues of TRIP8b (24). The *Prime* protein energy minimization module implemented in the Schrödinger suite was used to correct irrelevant side chains, add missing atoms, eliminate partial occupant rotamers, fix the undesired orientation of Asn, Gln, and His residues, and replace the “b” values by the optimized potential for liquid simulation (OPLS3) charges. The Glide (34) docking engine is built using a grid-based algorithm, and hence, a 12 × 12 × 12 Å grid cube was generated, which contained the aforementioned critical residues.

For the virtual screening, we used the curated library of approximately 11.2 million drug-like compounds described previously and the OPLS3 force field. The ligand Van der Waals radii was scaled to 0.80 Å with partial atomic charges < 0.15 esu and the three-tier Glide docking algorithm was executed. The output of this three-tier docking engine was analyzed using the XP-visualization tools by considering the interactions of the compounds with the critical residues reported previously. We selected the 77 compounds with a Glide docking score < −6.0. We then used an orthogonal docking engine (Surflex) implemented in the Sybyl interface of Tripos (35) to obtain consensus binding poses and to enrich the hit rate using alternative flexible ligand-docking tools (36). We found 52 compounds that showed similar binding poses with comparable binding scores by comparing the binding poses and scores obtained from both docking experiments. On the basis of commercial availability and synthetic tractability, we purchased 30 of these compounds for testing.

### Biochemical characterization of compound NUCC-0200590

**Figure 1:**
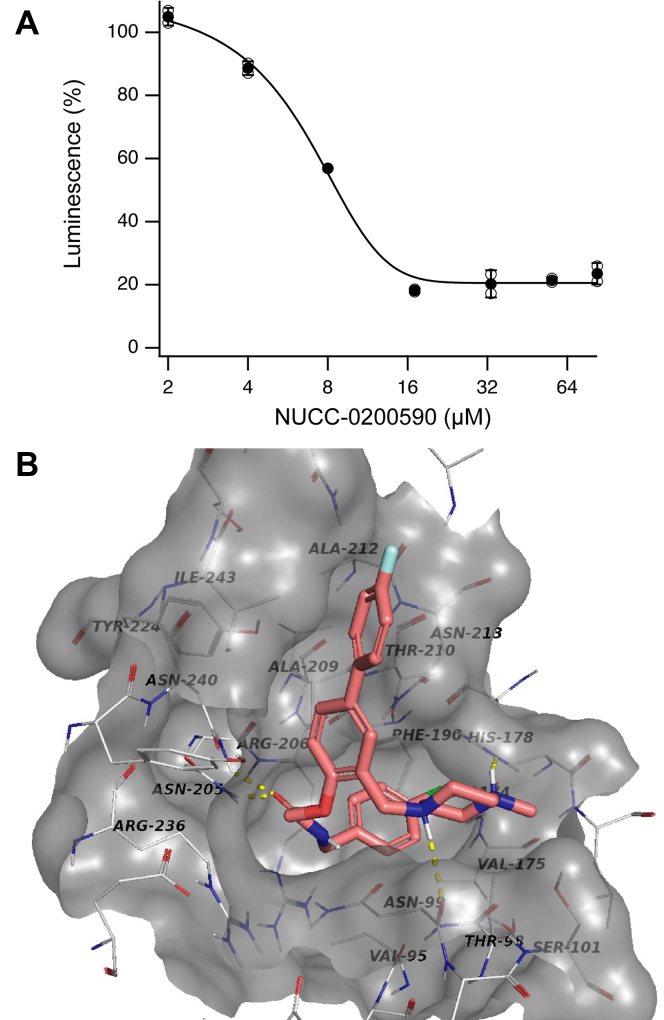
**NUCC-0200590 binds to TRIP8b and disrupts the TRIP8b-HCN interaction.***A*, AlphaScreen assay showing the ability of NUCC-0200590 to disrupt the interaction of TRIP8b–HCN1. NUCC-0200590 was incubated at varying concentrations with a peptide fragment corresponding to the C-terminal 40 amino acids of HCN1 and a large TRIP8b fragment described in Experimental procedures. *B*, docked pose of NUCC-0200590 to TRIP8b from our *in silico* screen with hydrogen bonds shown with *yellow dotted lines*. HCN, hyperpolarization-activated cyclic nucleotide–gated.

The 30 potential hits obtained from the aforementioned *in silico* screen were tested in biochemical assays to characterize biological activity. The molecular mechanism of HCN binding to TRIP8b has been elucidated by determination of the cocrystal structure of the carboxyl-terminal SNL sequence (SRLSSNL) of HCN2 bound to TRIP8b (24). The compounds were tested across a concentration range using our AlphaScreen assay (31) and compound NUCC-0200590 had an IC_50_ value of 7.0 ± 0.9 μM (Fig. 1*A*). The docked pose of NUCC-0200590 bound to TRIP8b is shown in Figure 1*B*.

### Synthesis of NUCC-0200590

We developed a synthetic route for compound NUCC-0200590 to prepare enough material needed for hit validation, *in vitro* and *in vivo* studies (Scheme S1). Alkylation of commercially available 5-bromosalicylaldehyde (1) with ethyl bromoacetate (2) afforded acetate 3 in quantitative yield. Reductive amination with *N*-methyl piperazine afforded four, which was hydrolyzed to carboxylic acid 5 under basic condition. Amide coupling of acid 5 with (4-chlorophenyl)methanamine using hydroxybenzotriazole and 1-ethyl-3-(3-dimethylaminopropyl)carbodiimide was not successful, so alternative approaches were explored. Though several early transition-metal complexes, especially from zirconium and titanium, have been used as catalysts in direct coupling of deactivated acids in high yields, the conversion was very low in our case. Conversion of the acid to an acid chloride before amidation was successful albeit with low yield. A larger set of coupling reagents were screened and O-(N-Succinimidyl)-N,N,N′,N′-tetramethyluronium tetrafluoroborate was identified as the best coupling reagent and produced the acetamide 6 in 66% yield. Suzuki coupling with (4-fluorophenyl)boronic acid afforded the final compound NUCC-0200590 in excellent yield. Conversion to the formic acid (FA) salt was achieved by exposure to the FA modifier in prep HPLC purification or to the hydrochloric acid (HCl) salt by treating the free base with 4M HCl in dioxane.

### Solubility of NUCC-0200590

We sought to validate the binding of NUCC-0200590 to TRIP8b using other biophysical assays. The free base of NUCC-0200590 has low solubility in PBS, which limits our ability to evaluate the binding to TRIP8b using other biophysical assays, such as saturation transfer difference (STD) NMR. To improve the aqueous solubility, we synthesized and characterized two salt forms of NUCC-0200590. The FA salt form was recovered after reverse-phase preparative HPLC while using 0.1% FA as a mobile phase modifier. Though the FA salt form had better aqueous solubility than the free base, it still required 15% dimethyl sufoxide (DMSO) in PBS to observe visible proton signals in ^1^H-NMR. On stirring the free amine in 4M HCl in dioxane, the compound was converted to the HCl salt as a white solid (Scheme S1). Comparison of the thermodynamic solubility of the three forms (free amine, FA salt, and HCl salt) of NUCC-0200590 performed with an in-house protocol (37) showed that the free amine had negligible solubility in PBS (pH 7.4) at room temperature (RT) after stirring for 16 h. The FA salt showed much improved solubility (45 μM) in PBS at pH 7.4 and even greater solubility (252 μM) was shown for the HCl salt. As a result, the HCl salt of NUCC-0200590 was used for biophysical assays.

### STD-NMR evaluation of NUCC-0200590 binding to TRIP8b

**Figure 2:**
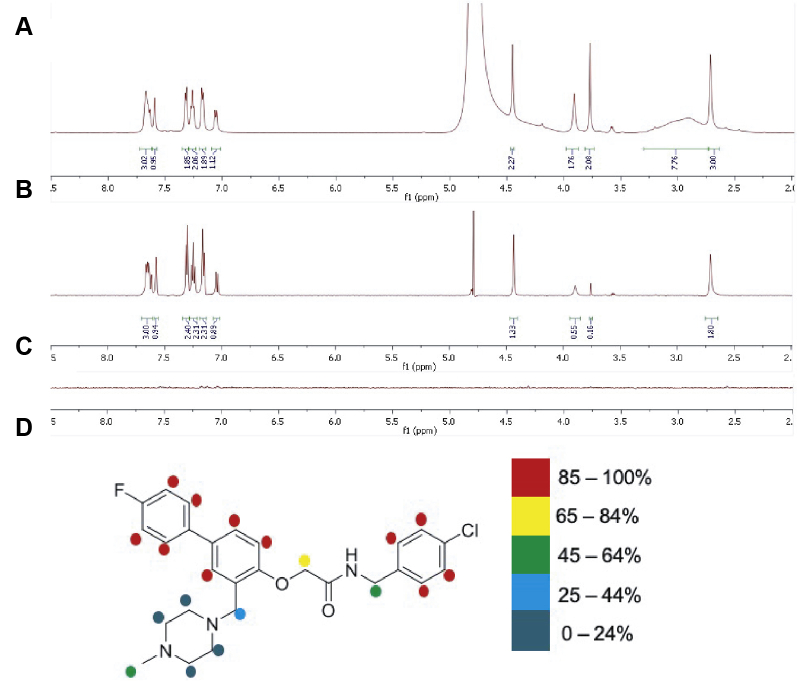
**Saturation-transfer difference NMR (STD-NMR) spectroscopy of NUCC-0200590 bound to TRIP8b.***A*, ^1^H-NMR reference spectrum of NUCC-0200590 in deuterated PBS. *B*, STD-NMR of NUCC-0200590 and TRIP8b in deuterated PBS. *C*, STD-NMR of NUCC-0200590 in deuterated PBS without TRIP8b. *D*, epitope mapping of NUCC-0200590 proton saturation showing the degree to which different protons are bound to TRIP8b protein.

To validate and gain structural understanding of the binding mode of NUCC-0200590 to TRIP8b, we performed STD-NMR experiments on the compound in the presence of TRIP8b. STD-NMR is a popular ligand-observed NMR technique used in studying protein–ligand interactions of weak affinity ligands (high nanomolar to low nanomolar) range. It is a robust technique because it relies on the measurement of relatively simple ^1^H-NMR signals of the ligand instead of the complex protein spectrum. This technique is based on the transfer of saturation from the protein to the bound ligand when the protein is selectively irradiated at a frequency where only resonances from the protein are located. The fast exchange of ligands with the binding sites allows for identification of the epitope of the protein surface close to the bound ligand. The STD-NMR of NUCC-0200590 bound to TRIP8b was initially carried out using a 20-fold ligand excess with respect to the protein at on-resonance frequency of −1 ppm and off-resonance frequency of 40 ppm. To better define the binding epitope, a 100-fold ligand excess was used (20 μM protein:2 mM ligand) in the STD-NMR experiment (Fig. 2). The reference ^1^H-NMR spectrum of 2 mM ligand in deuterated PBS at pH 7.4 showed the entire proton signals of the ligand (Fig. 2*A*). On irradiating a solution of NUCC-0200590 (2 mM) and TRIP8b (20 μM) at the on-resonance and off-resonance frequency, there was unambiguous evidence of saturation transfer to the ligand as shown by the STD-NMR (Fig. 2*B*). A control STD-NMR experiment of the ligand without the protein showed no ligand signal as there was no saturation transfer occurring due to the absence of TRIP8b (Fig. 2*C*). This confirmed the ability of NUCC0200590 to bind directly to TRIP8b. To get a structural understanding of the binding mode, the relative degree of saturation of the protons in the ligand was analyzed. Integration of the STD-NMR protons normalized to the protons at 7.65 ppm shows the aromatic protons with a 1:1 integral ratio in both the reference spectrum and the STD-NMR spectrum, which indicates that these protons are in close proximity to the protein. In comparison, integration of the aliphatic protons displayed integral ratio from 2:1 to 13:1 (reference spectrum: STD-NMR spectrum). This suggests that these protons were further away from the protein surface in comparison to the aromatic protons. Interestingly, the aliphatic protons of the piperazine showed no saturation transfer as they were not visible in the STD-NMR, indicating that they are not close to the binding pocket of TRIP8b, which agrees with the docked pose of NUCC-0200590 that shows this portion oriented toward the solvent. However, the methyl group on the piperazine showed significant evidence of saturation transfer, suggesting that the methyl group is near the protein surface possibly due to its angle of projection from the piperazine (Fig. 2*D*). Furthermore, the inhibitor was screened for *in vitro* stability toward mouse liver microsomes to evaluate its potential metabolism and suitability for future *in vivo* experiments. The microsomal stability was measured after a 60 min incubation at 37 °C with mouse liver microsomes. NUCC-0200590 was rapidly metabolized by mouse liver microsomes as only 3.7 % of the parent compound remained after incubation.

### NUCC-0200590 efficiently disrupts the TRIP8b–HCN interaction in cellular assays

We next turned our attention to testing the ability of NUCC-0200590 to disrupt the TRIP8b–HCN interaction in cell-based assays (using the free base version of the compound for the remainder of the paper). There are many isoforms of TRIP8b that differ in terms of the N-terminal exons that are included (with each isoform named by which exons are included (30)). As an exemplary TRIP8b isoform, we exclusively used the TRIP8b(1a-4) isoform, which is thought to be the most commonly occurring isoform in the brain (30). Thus, “TRIP8b” specifically refers to “TRIP8b (1a-4).” Similarly, although HCN1 and HCN2 have slightly different electrophysiological properties *in vitro* (9, 38), both are thought to bind to TRIP8b in precisely the same manner (30) and have a similar shift in their V_50_ when bound by TRIP8b. As such, we alternate between the two HCN isoforms (as outlined in the text) to confirm our results for both subunits in the experiments later. Thus, the phrase “HCN channels” refers to HCN1 and HCN2 channels collectively.

**Figure 3:**
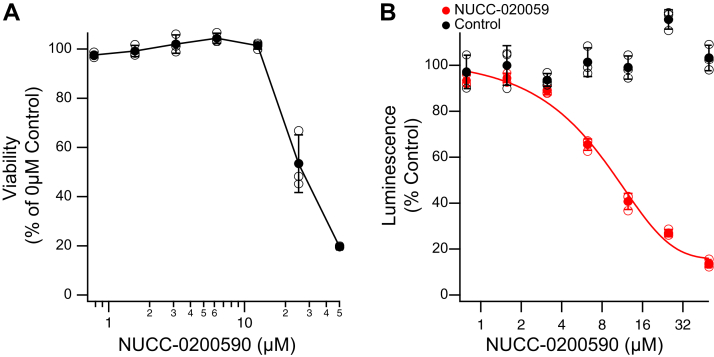
**Cellular toxicity of NUCC-0200590 and NanoBit assay confirming the ability of NUCC-0200590 disrupting the interaction in cells.***A*, HEK293T cells were incubated with varying concentrations of NUCC-0200590 (0–50 μM) for 24 h and then the viability assayed with a commercially available assay (see Experimental procedures). Data are expressed as a percentage of the viability in the 0 μM NUCC-0200590 control condition and error bars denote SEM. One-way ANOVA showed a main effect of concentration (F(6,20) = 138.84, *p* < 0.01). ∗denotes *p* < 0.05 on Tukey’s post hoc tests for pairwise comparisons of 25 μM condition with 0.8 μM to 12.5 μM conditions and pairwise comparisons of 50 μM condition with 0.8 μM to 12.5 μM conditions. *B*, HEK293T cells were transfected and 24 h later were incubated with NUCC-0200590 for 30 min. As a control (*black dots*), fragments of control proteins PRKACA and PRKAR2A were incubated with NUCC-0200590 for 30 min.

Having validated NUCC-0200590 as an inhibitor of the TRIP8b–HCN interaction in biophysical and biochemical assays, we tested its effects in several cellular assays. We first examined potential cytotoxicity by incubating HEK293T cells for 3 h or 24 h and analyzing cell viability. No cell death was observed after 3 h of incubation at all tested concentrations of NUCC-0200590 (data not shown). However, after 24 h incubation, at 25 μM and above, we noted cell death, but at lower concentrations there was no effect observed for NUCC-0200590 on cell viability (Fig. 3*A*). To study the ability of NUCC-0200590 to disrupt the TRIP8b–HCN interaction using full-length proteins, we first employed a NanoBiT split luciferase assay using full-length HCN1 and TRIP8b fused to the relevant Nanoluciferase protein fragments (referred to as SmBiT-HCN1 and LgBiT-TRIP8, see Experimental procedures). Briefly, the interaction of SmBiT-HCN1 with LgBiT-TRIP8b in live cells promotes assembly of functional luciferase through the proximity of the SmBiT and LgBiT fusion fragments, leading to the production of a luminescent signal in proportion to the amount of HCN1–TRIP8b binding (Fig. 3*B*). HEK293T cells were cotransfected with SmBiT-HCN1 and LgBiT-TRIP8b constructs, then pretreated for 30 min with varying concentrations of NUCC-0200590 before adding substrate and reading the luminescent signal. Increasing concentrations of NUCC-0200590 led to a reduction in HCN1–TRIP8b binding as indicated by normalized luminescence, with an IC_50_ of 5.62 ± 0.49 μM. To ensure the inhibition was specific, we also examined the interaction of the positive control pair protein kinase A catalytic (PRKACA) with type 2A regulatory subunit (PRKAR2A). In the presence of similar concentrations of NUCC-0200590, no change in the binding of these two proteins was observed (Fig. 3*B*), consistent with a specific effect of NUCC-0200590 on the TRIP8b–HCN interaction.

### NUCC-0200590 disrupts TRIP8b-mediated HCN channel gating and trafficking

**Figure 4:**
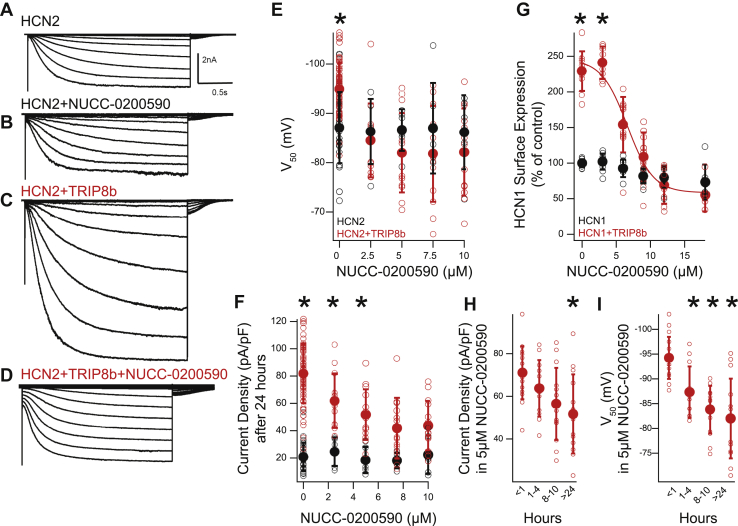
**NUCC-0200590 disrupts TRIP8b-mediated HCN channel regulation in HEK cells.***A*–*D*, representative whole-cell recordings were made from HEK cells stably expressing HCN2 and transiently transfected with an empty plasmid (*A* and *B*) or TRIP8b (*C* and *D*). Cells were also incubated for 24 h with NUCC-0200590 (*B* and *D*). *E*, the V_50_ of I_h_ was measured in the presence of increasing concentrations of NUCC-0200590 after incubation for 24 h. Note that the *y*-axis is negative such that more hyperpolarized values are higher up on the plot compared with less hyperpolarized values. At 0 μM NUCC-0200590, TRIP8b transfection hyperpolarized the V_50_ of I_h_ as has previously been shown. In the presence of NUCC-0200590, there was no effect of TRIP8b on the V50 of I_h_. two-way ANOVA of V_50_ with factors transfection (empty plasmid, TRIP8b) and drug concentration (0, 2.5, 5, 7.5, and 10 μM) showed no effect of transfection (F(1,147) = 1.27, *p* = 0.26) but an effect of drug (F(4,147) = 6.78, *p* = 0.0000485) and an interaction between the two conditions (F(4,147) = 5.34, *p* = 0.00047). Planned post hoc comparisons with Bonferroni correction for multiple comparisons (*p* < 0.01 significant) revealed a difference between the two transfection conditions only at 0 μM (t(78) = −4.9361, *p* = 0.000004) but not at other drug concentrations 2.5 μM (t(17) = 0.50, *p* = 0.62), 5 μM (t(18) = 1.31, *p* = 0.20), 7.5 μM (t(17) = 1.10, *p* = 0.28), or 10 μM (t(17) = 0.96, *p* = 0.34). For the HCN2 alone condition, n = 33, 6, 6, 6, and 6 cells for the 0, 2.5, 5, 7.5, and 10 μM condition, respectively, while for the HCN2 + TRIP8b condition n = 61, 13, 14, 13, and 13 cells. *F*, transfection with TRIP8b increased the surface expression of HCN channels and led to a higher density of I_h_, consistent with prior reports. However, increasing concentrations of NUCC-0200590 led to less current density of I_h_, indicating disruption of the TRIP8b–HCN interaction. Two-way ANOVA of current density with factors transfection (empty plasmid, TRIP8b(1a-4)) and drug concentration (0, 2.5, 5, 7.5, and 10 μM) showed an effect of transfection (F(1,148) = 90.76, *p* = 4.4712E-17), an effect of drug (F(4,148) = 7.74, *p* = 0.00001), and an interaction between the two (F(4,148) = 7.06, *p* = 0.00003). Planned post hoc comparisons with Bonferroni correction (*p* < 0.01 significant) revealed a difference between the two transfection conditions at 0 to 5 M drug concentrations 0 μM (t(78) = −13.41, *p*= 5.9243E-22), 2.5 μM (t(17) = −4.29, *p* = 0.0004), 5 μM (t(18) = −4.12, *p* = 0.0006), 7.5 μM (t(17) = −2.541, *p* = 0.021), or 10 μM (t(17) = −2.56, *p* = 0.019). For the HCN alone condition n = 26, 6, 6, 6, and 6 cells were used for the 0, 2.5, 5, 7.5, and 10 μM concentrations, respectively, while for the HCN + TRIP8b condition n = 54, 13, 14, 13, 14 cells were used. *G*, flow cytometry confirms that NUCC-0200590 disrupts TRIP8b-mediated HCN1 surface trafficking. Two-way ANOVA of surface HCN1 expression with factors transfection (empty plasmid, TRIP8b) and drug concentration (0, 3, 6, 9, 12, and 18 μM) showed an effect of transfection (F(1,98) = 7.73, *p* = 8.1086E-21), an effect of drug (F(5,98)= 64.668, *p* = 1.6692E-29), and an interaction between the two (F(5,98) = 36.37, *p* = 6.6418E-21). Planned post hoc comparisons with Bonferroni correction (*p* < 0.0083 significant) revealed a difference between the two transfection conditions at 0 to 9 μM drug concentrations 0 μM (t(20) = −15.8, *p*= 9.09E-13), 3 μM (t(17) = 17.68, *p* = 2.21E-12), 6 μM (t(17) = −4.26, *p* = 5.26E-4), 9 μM (t(20) = −5.93, *p* = 8.3E-6), 12 μM (t(14) = 0.99, *p* = 0.33), 18 μM (t(10) = 1.30, *p* = 0.22). For the HCN1 conditions, n = 12, 11, 8, 11, 8, 7 for 0, 3, 6, 9, 12, 18 μM conditions, respectively, while for the HCN1 + TRIP8b condition n = 10, 8, 11, 11, 8, 5. *H*, current density of I_h_ was investigated in HEK293 cells stably expressing HCN2 and transiently transfected with TRIP8b. Cells were recorded after varying amounts of time in 5 μM NUCC-0200590. One-way ANOVA revealed an effect of time (F(3,52) = 4.46, *p* = 0.007) and post hoc analysis revealed significant differences only when comparing less than 1 h with greater than 24 h in 5 μM NUCC-0200590. *I*, using the same cells collected for (*H*), we also analyzed the half activation potential (V_50_) of I_h_. One-way ANOVA revealed an effect of time (F(3,52) = 13.50, *p* = 1.24e-6) and post hoc analysis revealed differences between less than 1 h and 1 to 4 h (*p* = 0.013), between less than 1 h and 8 to 10 h (*p* = 4.38e-5), and between less than 1 h and greater than 24 h (*p* = 2.11e-6). For the <1 h condition, 16 cells were used, 1 to 4 h condition was 12 cells, 8 to 10 h condition was 14 cells, and over 24 h was 14 cells. HCN, hyperpolarization-activated cyclic nucleotide–gated.

To test whether the inhibition of TRIP8b–HCN binding by NUCC-0200590 influences HCN channel function, we performed whole-cell recordings using HEK293 cells stably expressing HCN2 (22). In these experiments, we were specifically interested in monitoring the two distinct effects of TRIP8b on HCN channel function as we reasoned that it may provide insight into the effect of NUCC-0200590. First, TRIP8b binding to HCN hyperpolarizes the V_50_ of I_h_ by ∼10 mV (22, 25, 29), indicating that a more hyperpolarized potential is needed to open the channel. Second, TRIP8b binding to HCN increases the surface expression of the channel so that there are more HCN channels present at the cell surface (greater I_h_ current density) (22, 25, 29). HEK293 cells stably expressing HCN2 (22) were transfected with either GFP and an empty vector or GFP and TRIP8b (Fig. 4 A-D). Twenty-four hours after transfection, the extracellular media was exchanged for media containing between 0 μM and 10 μM NUCC-0200590 (well below the toxic levels noted previously). After 24 h, whole-cell recordings were performed from GFP expressing cells. We first examined the V_50_ of I_h_ and observed a hyperpolarization of the V_50_ when HCN was coexpressed with TRIP8b (identical to prior work (22, 25, 29), Figure 4*E*). However, in the presence of 2.5 μM (and higher concentrations) of NUCC-0200590, there was no difference in the V_50_ between the TRIP8b condition and the control condition (Fig. 4*E*). Combined, these results indicate that NUCC-0200590 disrupts the hyperpolarizing effect of TRIP8b on the V_50_ of HCN channels and suggests that NUCC-0200590 causes TRIP8b to dissociate from HCN channels.

TRIP8b also upregulates HCN channel current density by increasing HCN channel surface trafficking (15, 23, 25, 39). Thus, we next examined the effect of NUCC-0200590 on HCN current density. As previously described, TRIP8b caused an increase in I_h_ current density in the absence of NUCC-0200590 (Fig. 4*F*), but incubation with increasing concentrations of NUCC-0200590 led to a dose-dependent reduction in I_h_ (Fig. 4*F*). Combined, these results establish that NUCC-0200590 abolished the known hyperpolarizing effect of TRIP8b on the V_50_ of HCN channels (suggesting TRIP8b is unbound from HCN) and disrupted TRIP8b-mediated increases in surface HCN channels (indicating fewer surface HCN channels).

To complement our electrophysiology results, we validated the effect of NUCC-0200590 on the surface trafficking of HCN channels using flow cytometry. In these assays, an HCN1 construct tagged with an extracellular hemagglutinin (HA) tag was used for flow cytometry such that surface expression of the channel can be measured in live cells (40). As has been shown previously, transfection of TRIP8b increased the surface expression of HCN1 (Fig. 4*G*) (40). Consistent with our electrophysiology results, incubation with NUCC-0200590 led to a dose-dependent reduction in surface expression of HCN1 with an IC_50_ of 6.81 ± 1.03 μM.

After noting that NUCC-0200590 disrupts the effect of TRIP8b on both HCN channel opening (V_50_) and surface trafficking, we next asked if these two effects are further dissociable. Specifically, we reasoned that looking at the time course of the change in V_50_ (TRIP8b binding to HCN channels) and I_h_ current density (surface expression of HCN channels) could inform the mechanism of NUCC-0200590’s action. Toward that end, we examined the effect of incubating 5 μM NUCC-0200590 on cells stably expressing HCN2 and transiently transfected with TRIP8b. We examined four time points: <1 h, 1 to 4 h, 8 to 10 h, and >24 h. Although it took at least 24 h for the current density (surface HCN channels) to become statistically different from the <1 h incubation condition, (Fig. 4*H*), the effect on V_50_ (indicating TRIP8b dissociation from HCN) was apparent at 1 to 4 h (Fig. 4*I*). These results indicate that NUCC-0200590 first disrupts the effect of TRIP8b on the V_50_ of I_h_ (within 1–4 h) prior to the loss of HCN channels from the cell surface. The implications of these findings for the mechanism of NUCC-0200590 are discussed later.

### NUCC-0200590 reduces sag ratio in CA1 pyramidal neurons of mouse brain slices

**Figure 5:**
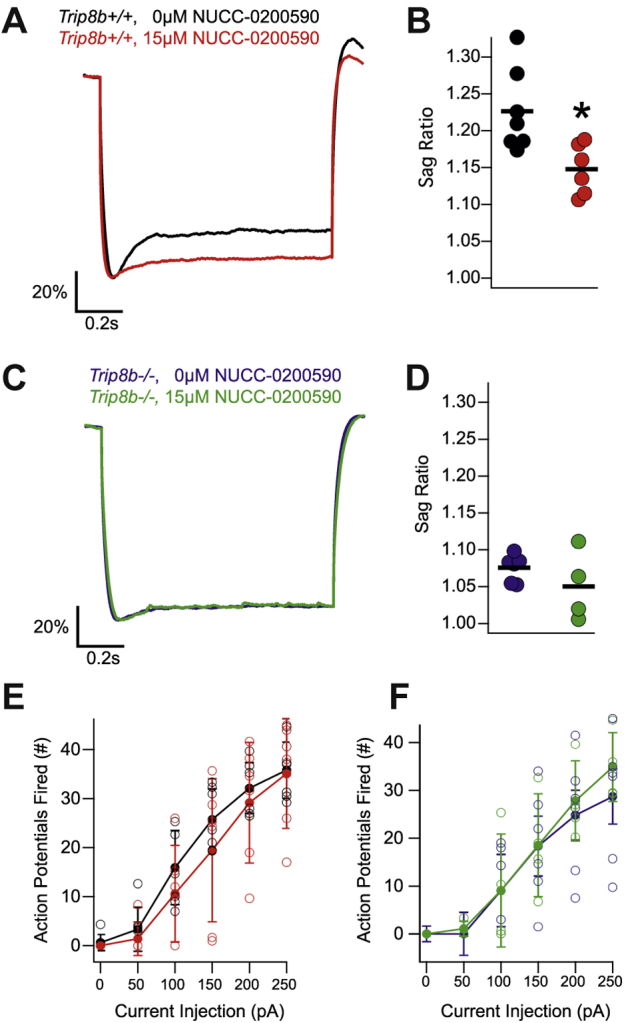
**NUCC-0200590 limits the current mediated by HCN channels in CA1 pyramidal neurons.***A*, whole-cell recordings were performed from CA1 pyramidal neurons in hippocampal slices made from *Trip8b*^*+/+*^ mice. A subset of slices were incubated with 15 μM NUCC-0200590 (in *red*). Representative current clamp traces are shown in response to a 1s long −200pA somatic current injection from a membrane potential of −70 mV. Note that traces are scaled so that the maximum deflection is 100% to facilitate comparison of the sag ratio. *B*, quantification of sag ratio in cells from *Trip8b*^*+/+*^ mice (0 μM/*black*: 1.22 ± 0.02, 15 μM/*red*: 1.14 ± 0.01, two tail *t* test yields t = 2.97, *p* = 0.0127, n = 7,6). *C*, whole-cell recordings performed from CA1 pyramidal neurons from *Trip8b*^*−/*−^ mice incubated with (*green*) or without (*blue*) 15 μM NUCC-0200590. *D*, no differences were noted on two tail *t* test comparing the sag ratio recorded from *Trip8b*^*−/*−^ cells (0 μM/*blue*: 1.07 ± 0.007, 15 μM/*green*: 1.05 ± 0.02, two tail *t* test yields t = 1.21, *p* = 0.25, n = 6,4). *E*, excitability of *Trip8b*^*+/+*^ CA1 pyramidal neurons was examined by measuring the number of action potentials fired in response to a 1s current injection of varying magnitude (see *x*-axis). No difference between the two conditions was noted using repeated measures ANOVA (F(5,55) = 0.78, *p* = 0.56, n = 7,6). *F*, the excitability of *Trip8b*^*−/*−^ CA1 pyramidal neurons was examined, but no difference between the two conditions was noted using repeated measures ANOVA (F(5,40) = 0.39, *p* = 0.85, n = 6,4). HCN, hyperpolarization-activated cyclic nucleotide–gated.

Having seen that NUCC-0200590 is effective *in vitro,* we next considered the effect of the compound on neurons *ex vivo*. We used 15 μM NUCC-0200590 (rather than 5 μM as in HEK cells in Fig. 4,*H* and *I*), knowing that slices are harder to penetrate and that they cannot be incubated *in vitro* for long as for HEK cells. We prepared slices from WT mice and incubated them for at least 2 h in 15 μM NUCC-0200590 before whole-cell recordings were made from CA1 pyramidal neurons. In the WT slices, incubation with NUCC-0200590 led to a reduction in the I_h_ sag ratio, a current clamp measurement of the activity of HCN channels (Fig. 5*A*/B). There were no differences noted in membrane resistance, resting membrane potential (Table S1), or excitability (Fig. 5*E*). To confirm that NUCC-0200590 limits the sag ratio by binding TRIP8b, we performed a control experiment using slices from genetic KO mice lacking all TRIP8b isoforms (Trip8b^*−/*−^) (15). Unlike the WT mice, there was no difference in sag ratio in the presence or absence of NUCC-0200590 in slices from Trip8b^−/−^ mice, consistent with a need for NUCC-0200590 to bind to TRIP8b in order to influence I_h_ (Fig. 5, *C*, *D* and *F*).

### NUCC-0200590 limits dendritic expression of HCN channels

**Figure 6:**
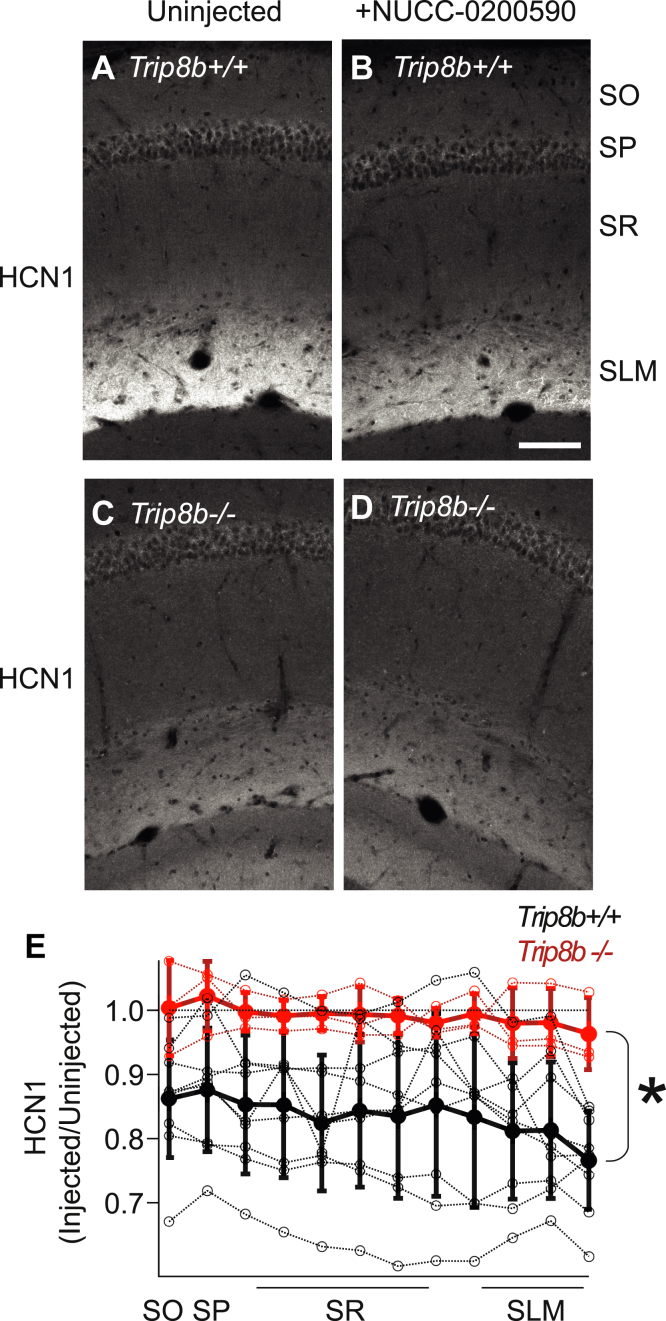
**Immunohistochemistry reveals that NUCC-0200590 causes a reduction in TRIP8b-mediated HCN channel trafficking.***A* and *B*, *Trip8b*^*+/+*^ mice were injected unilaterally with 1 μl of 10 μM NUCC-0200590 and then sacrificed for immunohistochemistry 48 h later. *C* and *D*, identical experiments were carried out in mice lacking TRIP8b (*Trip8b*^*−/*−^) to determine if the change in HCN1 expression could be attributed to NUCC-0200590 binding to TRIP8b. *E*, for quantification, regions of interest were drawn over the injected side (*B*) and scaled by the intensity of the staining of the contralateral vehicle-injected hemisphere (*A*) so that a value of ‘1’ reflects similar staining in the two hemispheres while values lower than 1 indicated less HCN1 expression. Quantification *Trip8b*^*+/+*^ (*black trace*) and *Trip8b*^*−/*−^ (*red trace*) mice unilaterally injected with 10 μM NUCC-0200590 and then taken for immunohistochemistry 48 h post injection. Individual mice are represented by *open circles* connected by *dotted lines* while the the mean is represented by a *filled circle* with a *solid line*. We observed a significant reduction in HCN1 staining in the injected hemisphere of *Trip8b*^*+/+*^ animals by repeated measures ANOVA (panel *C*, F(11,110) = 2.76, *p* = 0.003, n = 9 *Trip8b*^*+/+*^ mice, n = 3 *Trip8b*^*−/*−^ mice). The scale bar represents 100 microns. HCN, hyperpolarization-activated cyclic nucleotide–gated; SLM, Stratum lacunosum moleculare; SO, Stratum oriens; SP, stratum pyramidale; SR, stratum radiatum.

With data validating the effects on NUCC-0200590 in mouse brain slices, we next turned our attention to the question of whether NUCC-0200590 would disrupt TRIP8b function *in vivo*. TRIP8b plays an essential role in trafficking HCN channels into the distal dendrites of CA1 pyramidal neurons, and loss of TRIP8b′s function leads to fewer HCN channels in the distal stratum lacunosum moleculare (15). To circumvent the rapid metabolism of the compound (aformentioned), we intrahippocampally injected 1 μl of 10 μM NUCC-0200590 into the dorsal CA1 region of the hippocampus. The animals were sacrificed for immunohistochemistry to quantify HCN1 expression 48 h after the injection (see Experimental procedures). Compared with the contralateral hemisphere injected with an equivalent volume of vehicle (saline), the hemisphere injected with NUCC-0200590 showed significantly less staining for HCN1 (Fig. 6, *A* and *B*).

To confirm that the mechanism for the reduction in HCN1 staining was disruption of the TRIP8b-mediated interaction, we performed the same experiment in Trip8b^−/−^ mice. Importantly, although these animals lack TRIP8b-mediated HCN channel trafficking, they still express HCN channels in the distal dendrites, and these channels can be further reduced by expressing a TRIP8b mutant after viral manipulation (14). In contrast to the WT mice, Trip8b^−/−^ mice that were injected with NUCC-0200590 showed no further reduction in hippocampal HCN1 staining when taken for immunohistochemistry 48 h after injection (Fig. 6, *C*–*E*).

## Discussion

In this report, we have identified a new small molecule capable of disrupting the TRIP8b–HCN interaction. Several groups have suggested that the TRIP8b–HCN interaction may be a therapeutic target for the treatment of MDD (8, 12, 41) and the development of compounds such as NUCC-0200590 may lead to mechanistically distinct antidepressants. We demonstrated a strategy for the synthesis of this compound and established the efficacy of NUCC-0200590 in disrupting the TRIP8b–HCN interaction *in vitro* and *in vivo*. Direct binding of the compound to TRIP8b was established using STD-NMR, which also provides epitope mapping to show areas of the molecule that closely bind to the target protein (Fig. 2).

**Figure 7:**
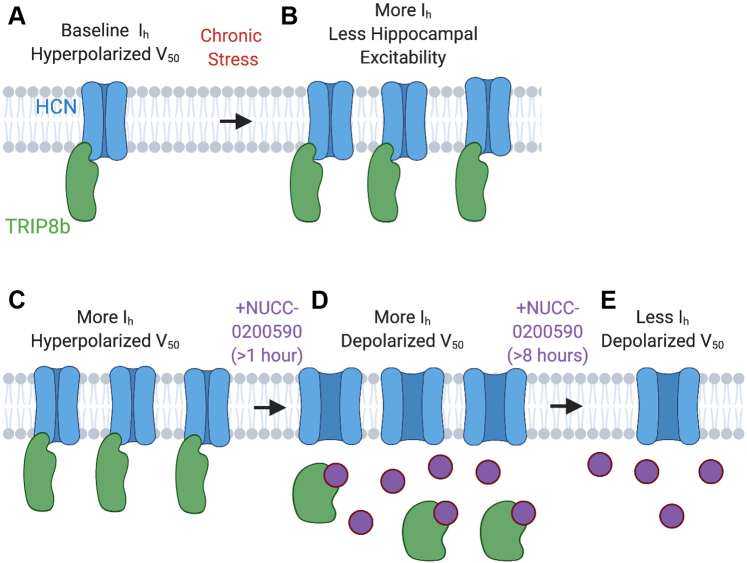
**Schematic highlighting proposed effect of NUCC-0200590 on TRIP8b-mediated HCN channel trafficking.***A*, model showing baseline levels of TRIP8b (*green*) bound to HCN (*blue*) with relative hyperpolarization of the V_50_ because of TRIP8b binding. *B*, summary of results from our prior report showing that after chronic stress, there is an increase in TRIP8b-mediated HCN channel surface expression in the dendrites of CA1 pyramidal neurons (17). The increase in surface expression of HCN channels causes the CA1 pyramidal neurons to be less excitable and ultimately influences cognitive impairment. *C*–*E*, interpretation of our results from HEK cell electrophysiology. *C*, overexpression of TRIP8b leads to high levels of surface HCN channel expression with hyperpolarized V_50_. *D*, after a brief incubation with NUCC-0200590 (*purple*), TRIP8b dissociates from HCN channels such that the V_50_ of HCN depolarizes. Given the short incubation period, the HCN channels remain at the surface of the cell (high current density) but are unbound by TRIP8b. This period is represented in our data by the 1 to 4 h incubations in Figure 4,*H* and *I*. *E*, after eight or more hours, the HCN channels at the cell surface that are no longer bound by TRIP8b are internalized and there is a reduction in I_h_ current density. This time point is represented in our data by the >24 h incubations in Figure 4,*H* and *I*. HCN, hyperpolarization-activated cyclic nucleotide–gated.

Our cell-based assays demonstrated that NUCC-0200590 effectively disrupts the TRIP8b–HCN interaction and provides an important mechanistic insight into how the compound limits TRIP8b-mediated HCN channel surface trafficking (Figs. 4 and 7). We observed that NUCC-0200590 limits the effect of TRIP8b on the V_50_ of HCN channels within 1 to 4 h, indicating that the drug is acting on TRIP8b–HCN complexes at the surface of the cell and causing a dissociation of TRIP8b from HCN (Fig. 7, *C* and *D*). While this result indicates that TRIP8b is likely dissociated from HCN (Fig. 7*D*), the channel remains present at the cell surface (given that we did not observe a statistically significant change in the surface density of I_h_ until a later time point, Fig. 4*H*). TRIP8b(1a-4) binding limits lysosomal degradation of the channel (13, 15, 39), a process potentially responsible for the reduction in HCN channel surface expression after treatment with NUCC-0200590 (Fig. 7*E*). Alternatively, it may be the case that the presence of NUCC-0200590 in complex with TRIP8b and HCN could promote an aberrant conformation of the TRIP8b–HCN channel complex that leads to degradation. Future efforts to characterize how NUCC-0200590 limits TRIP8b-mediated surface trafficking of HCN channels could yield a temporally precise method for disrupting HCN channel function that genetic knockdown strategies are not able to achieve.

The dorsal CA1 region plays an essential role in spatial memory and other forms of learning (42, 43) and several lines of evidence have shown that depression-like behaviors are associated with less excitable CA1 pyramidal neurons while antidepressant-like behaviors are associated with greater excitability (4, 5, 6, 7). Disrupting either of the two TRIP8b–HCN interacting sites is sufficient to limit TRIP8b-mediated HCN channel trafficking in neurons (14). Here, we have shown that NUCC-0200590, which only disrupts one of the two TRIP8b–HCN binding sites, is sufficient to limit TRIP8b-mediated HCN channel trafficking *in vivo* (Fig. 6). We recently demonstrated that chemogenetically limiting the TRIP8b–HCN interaction rescued the cognitive deficits that occurred in animals subjected to chronic stress (17); hence, we predict that a small molecule developed from NUCC-0200590 might be useful therapeutically to disrupt the TRIP8b–HCN interaction and treat the cognitive sequelae of chronic stress.

The biochemical observations made here suggest that a compound like NUCC-0200590 could meet an important unmet clinical need by functioning as a symptom-specific therapy for the treatment of MDD. The compound began disrupting the TRIP8b–HCN interaction in 1 to 4 h *in vitro* and led to reduced HCN surface trafficking noted afterward, suggesting that the compound could ultimately be used as a rapid acting therapy. This would provide a distinct advantage over existing monoaminergic therapies that typically require weeks to become efficacious. Interestingly, prior work has also shown that knockdown of HCN channel function in the dorsal hippocampus prior to stress limits the onset of behaviors relevant to MDD in an animal model (8). This raises the possibility that a therapy such as NUCC-0200590 could be used prophylactically to prevent the onset of a major depressive episode or to prevent the development of MDD entirely.

A significant limitation of NUCC-0200590 is the compound’s rapid degradation by liver microsomes. This liability will need to be addressed with additional chemical modifications to enable its use in animal models relevant to MDD. To date, most work on the TRIP8b–HCN interaction *in vivo* has been carried out using genetic tools, but future efforts may take advantage of NUCC-0200590 or a derivative to better characterize the precise half-life of HCN channels at the neuronal surface *in vivo* and in distinct molecular contexts. The TRIP8b–HCN interaction holds promise as a mechanistically distinct therapeutic target for the treatment of MDD. While more work is needed to create a small molecule candidate for human trials, compounds acting on this interaction may ultimately have use for a variety of additional disorders including chronic pain (44) and anxiety (8, 45).

## Experimental procedures

### Chemistry

Unless otherwise noted, all materials for the synthetic chemistry portion were obtained from commercial suppliers (Combi-Blocks, CombiPhos, Fisher Scientific, Sigma–Aldrich, or VWR) and used without further purification. The ^1^H-and ^13^C-NMR spectra were recorded on a Bruker AVANCE 500 MHz spectrometer using CDCl_3_, CD_3_OD, or D_2_O as the solvent. Chemical shifts are expressed in ppm (δ scale) and referenced to residual protonated solvent. When peak multiplicities are reported, the following abbreviations are used: s (singlet), d (doublet), t (triplet), q (quartet), m (multiplet), or br s (broad singlet). TLC was performed on glass backed Merck silica gel 60 F_254_ plates, column chromatography was performed using KP-SIL silica gel (Biotage), and flash column chromatography was performed on Biotage prepacked columns using the automated flash chromatography system Biotage Isolera One. Low resolution LC-MS was performed on a Waters Acquity-H UPLC/MS system with a 2.1 mm × 50 mm, 1.7 μm, reversed phase BEH C18 column and LC-MS grade solvents. A gradient elution from 95% water + 0.1% FA/5% acetonitrile + 0.1% FA to 95% acetonitrile + 0.1% FA/5% water + 0.1% FA over 2 min plus a further minute continuing this mixture at a flow rate of 0.85 ml/min was used as the eluent. Total ion current traces were obtained for electrospray ionization (ESI), positive and negative ionization (ESI+/ESI-). The purities of all the final compounds were of >95% as determined by ultra-performance liquid chromatography analysis unless otherwise indicated. High-resolution mass analysis was performed on Agilent 6210A LC-TOF. Preparative HPLC purification was carried out using a Gilson GX-271 preparative scale reverse phase HPLC system with Gilson model 159 UV–VIS detector and Phenomenex Kinetex 5 μm, C18, 100 A, 50 mm × 30 mm column. Compounds were eluted using a gradient elution of A:B (90:10–0:100) over 5 min at a flow rate of 50.0 ml/min, where solvent A was H_2_O (with 0.1% FA) and solvent B was CH_3_CN (with 0.1% FA). The product fractions were combined and dried using a Genevac EZ-2 Centrifugal Evaporator.

### Ethyl 2-(4-bromo-2-formylphenoxy)acetate (3)

The compound ethyl 2-(4-bromo-2-formylphenoxy)acetate was synthesized according to Org. Lett., 2015, 17 (23), pp 5824 to 5827.

### Ethyl 2-(4-bromo-2-((4-methylpiperazin-1-yl)methyl)phenoxy)acetate (4)

A mixture of ethyl 2-(4-bromo-2-formylphenoxy)acetate (0.70 g, 2.44 mmol), 1-methylpiperazine (0.295 ml, 2.68 mmol), and acetic acid (0.140 ml, 2.44 mmol) in 1,2-dichloroethane (10 ml) was stirred at RT for 30 min, then sodium triacetoxyhydroborate (0.62 g, 2.93 mmol) was added and stirring was continued for 5 h. The reaction was diluted with dichloromethane (DCM), quenched with NaBHCO_3_, and extracted with DCM (10 ml × 3). The organic layer was dried with Na_2_SO_4_, concentrated, and was purified by Biotage eluting with 6% MeOH in DCM to afford 4 (0.73 g, 81%) as a colorless oil. ^1^H NMR (500 MHz, CDCl_3_) δ 7.26 (s, 1H), 7.01 (dd, *J* = 8.7, 2.6 Hz, 1H), 6.37 (d, *J* = 8.7 Hz, 1H), 4.35 (s, 2H), 3.98 (q, *J* = 7.1 Hz, 2H), 3.34 (s, 2H), 2.58 to 2.26 (m, 4H), 2.26 to 2.08 (m, 4H), 2.03 (s, 3H), and 1.02 (t, *J* = 7.1 Hz, 3H). ^13^C NMR (126 MHz, CDCl_3_) δ 168.41, 155.15, 132.98, 130.37, 129.73, 114.07, 113.50, 65.92, 61.21, 55.40, 55.09, 52.89, 45.95, and 14.10. LC-MS (ESI) m/z: [M + H]^+^ 371.3.

### 2-(4-Bromo-2-((4-methylpiperazin-1-yl)methyl)phenoxy)acetic acid (5)

To a solution of ethyl 2-(4-bromo-2-((4-methylpiperazin-1-yl)methyl)phenoxy)acetate (0.62 g, 1.67 mmol) in tetrahydrofuran (6 ml) and water (2 ml) was added lithium hydroxide monohydrate (0.21 g, 5.01 mmol), and the reaction was stirred at RT until complete while monitored by TLC. The solvent was removed *in vacuo* to yield a crude solid that contained the lithium salt. The crude solid was dissolved in DCM, filtered, and the filtrate was purified by prep HPLC and concentrated to afford 5 (0.57 g, 100%) as a white fluffy solid. ^1^H NMR (500 MHz, CDCl_3_) δ 10.56 (s, 2H, COOH), 8.29 (s, 2H, FA), 7.33 (d, *J* = 8.7 Hz, 1H), 7.22 (s, 1H), 6.74 (d, *J* = 8.6 Hz, 1H), 4.50 (s, 2H), 3.50 (s, 2H), 3.23 to 2.77 (m, 5H), 2.79 to 2.64 (m, 4H), and 2.61 (s, 3H). ^13^C NMR (126 MHz, CDCl_3_) δ 172.28, 166.94, 155.23, 134.55, 132.42, 127.20, 114.04, 113.95, 67.19, 57.15, 52.86, 50.25, and 43.26. LC-MS (ESI) m/z: [M + H] + 343.3.

### 2-(4-Bromo-2-((4-methylpiperazin-1-yl)methyl)phenoxy)-N-(4-chlorobenzyl)acetamide (6)

To a solution of 2-(4-bromo-2-((4-methylpiperazin-1-yl)methyl)phenoxy)acetic acid (0.30 g, 0.87 mmol) and (4-chlorophenyl)methanamine (0.13 ml, 1.05 mmol) in dimethylformamide (4 ml) was added 2-(2,5-dioxopyrrolidin-1-yl)-1,1,3,3-tetramethyluronium tetrafluoroborate (0.53 g, 1.75 mmol) and *N*-ethyl-*N*-isopropylpropan-2-amine (0.46 ml, 2.62 mmol). The reaction was stirred at RT overnight. The reaction was quenched with water and extracted with ethyl acetate (EA; 20 ml × 3). The organic layers were combined, washed with brine, dried with Na_2_SO_4_, and concentrated *in vacuo*. The crude was purified by prep HPLC and concentrated to afford 6 (0.27 g, 66%) as a yellow solid. ^1^H NMR (500 MHz, CDCl_3_) δ 8.24 (s, 1H, FA), 7.46 (d, *J* = 8.7 Hz, 1H), 7.36 (s, 1H), 7.33 to 7.26 (m, 2H), 6.99 (dd, *J* = 8.4, 3.6 Hz, 2H), 6.88 to 6.81 (m, 1H), 4.73 (d, *J* = 3.3 Hz, 2H), 4.62 to 4.53 (m, 2H), 3.49 (d, *J* = 3.5 Hz, 2H), 2.65 to 2.41 (m, 4H), and 2.41 to 2.04 (m, 7H). ^13^C NMR (126 MHz, chloroform-*d*) δ 168.89, 155.09, 136.79, 134.55, 133.13, 132.01, 128.81, 128.22, 113.92, 113.45, 67.19, 58.53, 54.80, 53.47, 45.93, and 41.95. LC-MS (ESI) m/z: [M + H] + 457.3.

### N-(4-chlorobenzyl)-2-((4'-fluoro-3-((4-methylpiperazin-1-yl)methyl)-[1,1'-biphenyl]-4-yl)oxy)acetamide (NUCC-0200590)

To a solution of 2-(4-bromo-2-((4-methylpiperazin-1-yl)methyl)phenoxy)-N-(4-chlorobenzyl)acetamide (0.18 g, 0.38 mmol), (4-fluorophenyl)boronic acid (0.08 g, 0.57 mmol), and K_2_CO_3_ (0.16 g, 1.13 mmol) in dioxane (2 ml) and water (1 ml) was added PdCl2(dppf) (0.03 g, 0.04 mmol) and degassed with N_2_ for 5 min. The reaction vial was sealed and heated to 100 °C for 1 h. The reaction was cooled to RT and extracted with EA (5 ml × 3). The organic layers were combined, washed with brine, dried with Na_2_SO_4_, and concentrated *in vacuo*. The crude was purified with Biotage eluting with 50% EA in hexanes to afford NUCC-0200590 (0.13 g, 73%) as a thick yellow oil. ^1^H NMR (500 MHz, CD_3_OD) δ 7.63 to 7.54 (m, 4H), 7.29 to 7.25 (m, 2H), 7.21 to 7.17 (m, 2H), 7.15 (d, *J* = 8.7 Hz, 2H), 7.10 (d, *J* = 9.2 Hz, 1H), 4.78 (s, 2H), 4.47 (s, 2H), 3.89 (s, 2H), 2.98 to 2.77 (m, 4H), 2.77 to 2.58 (m, 4H), and 2.45 (s, 3H). ^13^C NMR (126 MHz, CD_3_OD) δ 171.45, 163.67 (d, *J* = 249.89 Hz), 157.23, 138.50, 137.66 (d, *J* = 3.21 Hz), 135.29, 134.05, 131.54, 130.01, 129.64, 129.51 (d, *J* = 7.23 Hz), 129.34, 116.57 (d, *J* = 23.46 Hz), 114.27, 68.53, 57.72, 54.42, 52.61, 44.88, and 43.01. HRMS *m/z* calculated for C_22_H_29_F_3_N_4_O [M + H]^+^: 481.1940; found: 481.1937.

### N-(4-chlorobenzyl)-2-((4'-fluoro-3-((4-methylpiperazin-1-yl)methyl)-[1,1'-biphenyl]-4-yl)oxy)acetamide formate (NUCC-0200590 FA salt)

The crude of the free NH_2_ of NUCC-0200590 was subjected to purification by prep HPLC and concentrated to give the FA salt. ^1^H NMR (500 MHz, CD_3_OD) δ 8.52 (s, 1H, FA), 7.61 to 7.53 (m, 4H), 7.26 (d, *J* = 8.4 Hz, 2H), 7.18 (d, *J* = 8.5 Hz, 2H), 7.14 (d, *J* = 8.7 Hz, 2H), 7.10 to 7.06 (m, 1H), 4.77 (s, 2H), 4.47 (s, 2H), 3.86 (s, 2H), 2.92 to 2.76 (m, 4H), 2.76 to 2.60 (m, 4H), 2.44 (s, 3H). ^13^C NMR (126 MHz, CD_3_OD) δ 171.42, 163.74 (d, *J* = 248.18 Hz), 157.21, 138.51, 137.68 (d, *J* = 3.17 Hz), 135.25, 134.04, 131.51, 129.98, 129.64, 129.50 (d, *J* = 8.22 Hz), 129.26, 125.87, 116.56 (d, *J* = 21.85 Hz), 114.22, 68.54, 57.72, 54.44, 52.57, 44.82, and 42.98.

### N-(4-chlorobenzyl)-2-((4′'-fluoro-3-((4-methylpiperazin-1-yl)methyl)-[1,1′'-biphenyl]-4-yl)oxy)acetamide hydrochloride (NUCC-0200590 HCl salt)

To a solution of NUCC-0200590-free base (0.10 g, 0.21 mmol) in dioxane was added 4M HCl in dioxane (0.2 ml, 0.62 mmol). The solution was stirred at RT for 1 h. The solution was concentrated and triturated with ether to afford NUCC-0200590 (HCl salt) (0.11 g, 94%) as a white solid. ^1^H NMR (500 MHz, 126 MHz, D_2_O) δ 7.76 (dd, *J* = 8.6, 2.4 Hz, 1H), 7.70 to 7.63 (m, 3H), 7.33 to 7.29 (m, 2H), 7.29 to 7.24 (m, 2H), 7.19 (dd, *J* = 8.7, 3.2 Hz, 3H), 4.96 (s, 2H), 4.48 (s, 2H), 4.43 (s, 2H), 3.78 to 3.29 (m, 8H), and 2.96 (s, 3H). ^13^C NMR (126 MHz, D_2_O) δ 170.53, 162.97, 161.02, 155.36, 136.58, 134.77 (d, *J* = 2.95 Hz), 133.64, 132.14, 131.21, 129.89, 128.60, 128.21, 128.08 (d, *J* = 8.09 Hz), 117.74, 115.49 (d, *J* = 23.39 Hz), 113.02, 67.09, 55.79, 50.09, 48.50, 42.84, and 42.04.

### AlphaScreen assay

Twenty nanomolar of GST-tagged HCN1_c40_ protein (corresponding to the C-terminal 40 amino acids of HCN1) was incubated with 200 nM of His-tagged TRIP8b (241–602) and varying concentrations of the test compound as described in our prior report (31, 46). After a 3 h incubation, anti-GST AlphaScreen acceptor beads were added for 1.5 h, followed by AlphaScreen nickel chelate donor beads for 1.5 h. AlphaScreen signal was quantified using a PerkinElmer EnSpire multimode plate reader.

### STD-NMR

STD NMR spectrum was recorded using a Bruker Advanced 500 spectrometer equipped with a Cryogenic probe and processed by Bruker Topspin software. An experiment to confirm TRIP8b binding of the inhibitor consisted of compound NUCC-0200590 (100 μM) and TRIP8b(1a-4) (5 μM) in 5% DMSO-d_6_ and deuterated PBS, pH 7.4 buffer (600 μl total volume). The prepared solution was vortexed for 30 s and submitted for NMR. The epitope mapping experiment was performed the same way, except using 2 mM NUCC-0200590 and 20 μM TRIP8b to increase signal to noise. STD-NMR experiments were performed with a train of 50 ms Gaussian-shaped saturating pulses at 200 Hz power for 2 s with “on” resonance saturation at −1 ppm and “off” resonance saturation at 40 ppm. The relaxation delay was 2 s before the saturating pulses. The number of scans was 2048, and the spectral width was 10 ppm.

### Cell viability assays

HEK293T cells (ATCC) were maintained as previously described (22). These cells were plated at 30 to 50% confluence and incubated for 24 h with the concentration of NUCC-0200590 specified in the main text. The cells were then incubated with alamarBlue assay reagent per the manufacturer’s instructions (ThermoFisher) prior to reading the plates on a plate reader.

### NanoBiT live cell protein–protein interaction assay

HEK293T cells (obtained freshly from ATCC) were plated in Dulbecco's modified Eagle's medium (Thermo Fisher; #11965-092) with 10% fetal bovine serum (Thermo Fisher; #16000-044), and Pen/Strep (Thermo Fisher; #15140-122) at one million cells per well in 6-well tissue culture–treated plates. The next day, cells were cotransfected using Lipofectamine 3000 with plasmids encoding the small Nanoluciferase fragment (SmBiT) fused to the N terminus of full-length HCN1 (SmBiT-HCN1) and the large Nanoluciferase fragment (LgBiT) fused to the N terminus of TRIP8b(1a-4) (LgBiT-TRIP8b). The plasmids were constructed by cloning into backbone vectors from the Promega NanoBiT MCS cloning kit #N2014. As a control for specific inhibition, other cells were cotransfected with SmBiT-PRKACA and LgBiT-PRKAR2A positive control plasmids from the kit. Twenty-four hours post-transfection, the cells were trypsinized with TrypLE (Thermo Fisher; #12604-013), washed, and suspended in phenol red-free Opti-MEM I (Thermo Fisher; #11058-021), and 25,000 cells per well were plated in white 384-well tissue culture–treated plates (Greiner; #781080). A dilution series of NUCC-0200590 or DMSO vehicle control was added to each well and incubated for 30 min at 37 °C. Luminescence was generated by adding Nano-Glo live cell substrate (Promega; #N2011) and incubating for 30 min at 37 °C prior to detecting on a luminescent reader (Biotek Neo2).

### Flow cytometry

The surface HCN expression evaluation by flow cytometry was performed as described previously (40). HEK293 cells were transfected with HA-HCN1 and TRIP8b(1a-4) or pEGFP (as a control). Cells were incubated with varying concentrations (in μM: 3, 6, 9, 12, and 18) of NUCC-0200590 or DMSO (as a control) overnight. Nonpermeabilized cells were stained with mouse anti-HA primary antibody to label surface HA and then were stained with secondary antibody conjugated to Alexa-647 (Invitrogen). Cells were run in an LSR Fortessa flow cytometer (BD Biosciences) at the Vanderbilt University Medical Center Flow Cytometry Core, and data were analyzed by FlowJo software (BD Biosciences). The presented fluorescence index was calculated as the integral of the Alexa-647 fluorescence with respect to cell number, which was normalized by enhanced GFP (eGFP)–positive cells in each transfection condition. The fluorescence indices for each condition were normalized to the control transfection condition (HCN1 plus eGFP). Statistical analyses were performed using one-way ANOVA with Tukey’s post hoc test.

### HEK electrophysiology

HEK cells stably expressing HCN2 (described in our previous report (22)) were plated on autoclaved 12 mm diameter glass coverslips coated with poly-L-lysine (0.1 mg/ml) and washed with standard Hank’s Balanced Salt medium (without calcium or magnesium). Cells were transiently transfected 24 h later with EGFP plus a control vector or TRIP8b(1a-4) using Lipofectamine 2000 according to the manufacturer’s instructions. Lipofectamine exposure was limited to 60 min, followed by rinsing and replacing with of preconditioned culture medium. Different doses of NUCC-0200590 were added in the medium following transfection as described in Results. For time course studies, 5 μM of NUCC-0200590 was added in the medium. Whole-cell recordings were performed 24 to 48 h post-transfection with pipettes made from borosilicate glass using a vertical puller (Narishige) with a final resistance of ∼3 MΩ. Cells were held at −40 mV in voltage clamp and stepped from −40 to −120 mV in 10 mV increments. Extracellular solution consisted of (in mM): 145 NaCl, 10 KCl, 10 glucose, 10 Hepes, 2 CaCl_2_, 1 MgCl_2_, buffered to pH 7.4, and an osmolarity of 312 to 315 mOsm. Intracellular pipette solution contained 135 K-gluconate, 10 MgCl_2_, 0.1 CaCl_2,_ 1 EGTA, 10 Hepes, and 2 Mg-ATP buffered to pH 7.3 and an osmolarity of 295 to 305 mOsm. Maximal tail current amplitudes were fitted to the Boltzmann equation as described previously (25). Maximal I_h_ current density was calculated by dividing the maximal tail current by the cell capacitance, as described previously (24). Currents were recorded *via* WinWCP software (University of Strathclyde), a MultiClamp 700A amplifier (Molecular Devices), and a National Instruments USB6221 interface card. Sampling rate was 10 kHz. Series resistance was monitored throughout each experiment and cells were discarded if the series resistance rose during the experiment. Statistical analyses were performed using one-way ANOVA with Tukey’s post hoc test.

### Animals

Six to eight weeks old animals were used in this study with all experiments conducted between 8 AM and 5 PM. Trip8b^−/−^ mice were bred as heterozygotes on a C57BL/6J background as previously described (15). All animal protocols were approved by the IACUC committees of Vanderbilt University Medical Center.

### Slice electrophysiology

Whole-cell recordings from CA1 pyramidal neurons were performed using an electrophysiology rig described previously (17). Three hundred micrometer thickness sagittal slices were made from male and female mice. Artificial cerebrospinal fluid was composed of 125 mM NaCl, 2.5 mM KCl, 25 mM NaHCO3, 1.25 mM Na_2_PO_4_, 1 mM MgCl_2_, 2 mM CaCl_2_, and 25 mM glucose while the internal solution was 115 mM K-gluconate, 20 mM KCl, 10 mM Na_2_ phosphocreatine, 2 mM MgATP, and 0.3 mM NaGTP. NUCC-0200590 was incubated in the slice chamber in a subset of experiments as described in the text. With 5 μM CGP52432, 2 μM SR-95531, and 10 μM MK-801 in artificial cerebrospinal fluid to block GABA_B_R_s_, GABA_A_Rs, and NMDARs, neurons were maintained at −70 mV in current-clamp mode. Step currents were injected from −200 to 250 pA (1000 ms duration) with a 10 s interval between steps. Data were collected with a Multiclamp 700B amplifier (filtered at 2 kHz) and a Digidata 1440A (digitized at 20 kHz) (Molecular Device Inc) and analyzed with Clampfit 10.0 (Molecular Device Inc).

### Intrahippocampal NUCC-0200590 injections

The stereotaxic injections were performed with minimal modifications from our previous work (17). Mice aged 6 to 8 weeks old were anesthetized with 1 to 2% inhaled isoflurane and mounted on a stereotaxic apparatus (Stoelting). A small incision was made to visualize bregma and lambda and a craniotomy was made with a dental drill. A Hamilton syringe was then lowered to the following coordinates: 2.3 mm A/P, ±1.3 mm M/L, −1.7 mm D/V. The syringe was held there for 2 min prior to injecting 1 μl of 10 uM of NUCC-0200590 (or saline control) at a rate of 0.3 μl/min. The syringe was then held in place for an additional 5 min before the syringe was slowly withdrawn.

### Immunohistochemistry

Immunohistochemistry was performed as described in a prior report (17) using primary antibodies: anti-HCN1, anti-HCN2, and anti-TRIP8b. Images were acquired using a confocal microscope at the Vanderbilt Cell Imaging Shared Resource supported by 10.13039/100000002NIH grants (CA68485, DK20593, DK58404, DK59637, and EY08126) and then analyzed using custom-written routines in MATLAB (Mathworks). The analysis of immunohistochemical images was carried out identically to our previous report (14), and a blinded observer drew regions of interst (ROIs) over the *stratum oriens* and *stratum pyramidale*. A larger ROI was also drawn encompassing the *stratum lacunosum moleculare* and *stratum radiatum*, which was then subdivided into ten equally spaced ROIs to examine the distal dendritic enrichment of HCN channels.

## Data analysis

Statistical tests are identified throughout the article with ∗ indicating a statistically significant result. Error bars represent SD and *open circles* indicate individual data points while *filled circles* represent the mean of that experimental condition.

Schematic of TRIP8b-mediated HCN channel trafficking was created using BioRender.com

## Data availability

All data described are located in this article.

## Supporting information

This article contains supporting information.

## Conflict of interest

The authors declare that they have no conflicts of interest with the contents of this article.
