# Supplementary material for: Discovery of a small-molecule inhibitor of the TRIP8b–HCN interaction with efficacy in neurons
Source: J Biol Chem. 2022 May 24;298(7):102069. doi: 10.1016/j.jbc.2022.102069 (PMC9243175; doi:10.1016/j.jbc.2022.102069)
Supplement: Supplemental Information [file mmc1.docx]

**Supporting information for:**

**Discovery of a small-molecule inhibitor of the TRIP8b-HCN interaction with efficacy in neurons**

Ye Han^1^, Iredia D. Iyamu^2^, Matthew R. Clutter^3^, Rama K. Mishra^4^, Kyle A. Lyman^5^, Chengwen Zhou^1^, Ioannis Michailidis^1^, Maya Y. Xia^1^, Horrick Sharma^2^, Chi-Hao Luan^3,7^, Gary E. Schiltz^6,7,8‡^, Dane M. Chetkovich^1‡^

**
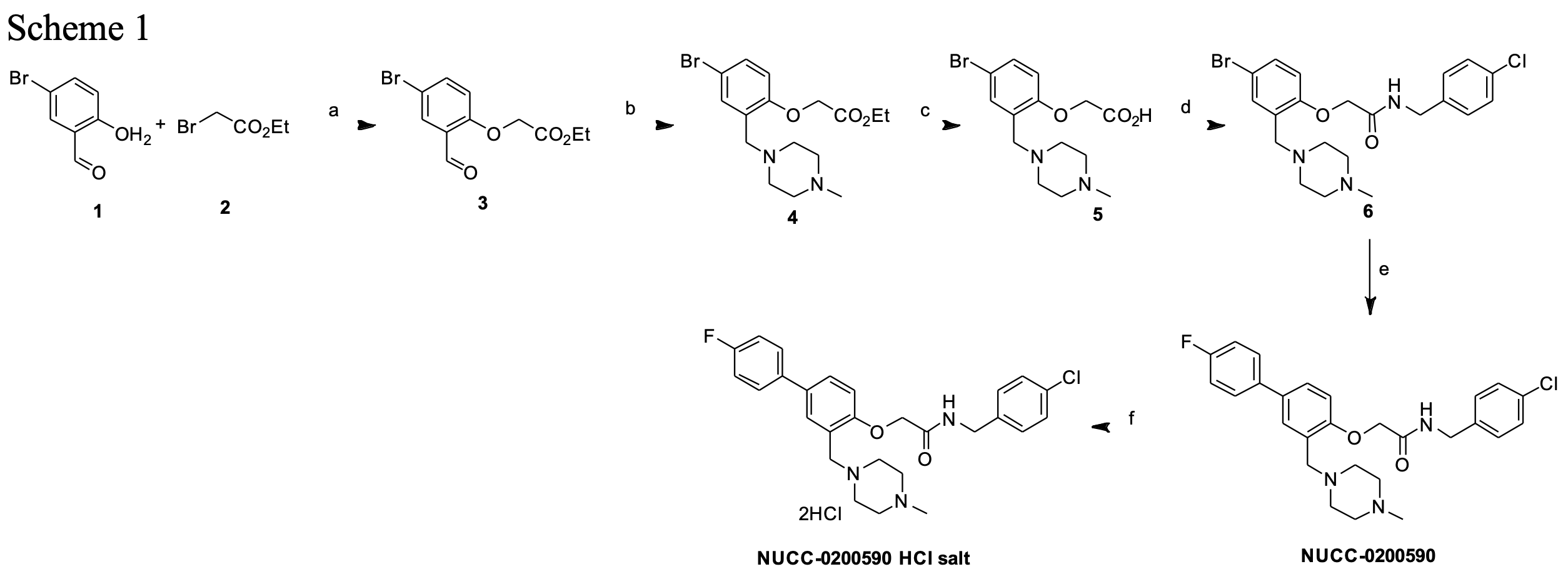
**

**Scheme 1)** Reaction conditions (a) Cs_2_CO_3_, DMF, rt, 1 h, quant.; (b) 1-methylpiperazine, NaBH(OAc)_3_, AcOH, 1,2-DCE, 5 h, 81%; (c) LiOH, THF:H_2_O = 3:1, rt, quant.; (d) (4-chlorophenyl)methanamine, TSTU, DIPEA, DMF, rt, overnight, 66%; (e) (4-fluorophenyl)boronic acid, PdCl_2_(dppf), K_2_CO_3_, dioxane:H_2_O = 2:1, 100 ^o^C, 1 h, 73%; (f) 4M HCl/dioxane, 1 h, rt, 94%.

**Table 1)** *Descriptive statistics for CA1 pyramidal neurons recorded from Trip8b^+/+^ and Trip8b^-/-^ slices. Mean is displayed with standard error of the mean in parentheses. P Values represent the result of 2 tailed T tests.*

| ***Trip8b+/+*** | 0µM | 15µM | P Value |
| --- | --- | --- | --- |
| Membrane Resistance (MΩ) | 89.1 (8.8) | 90.55(13.6) | 0.92 |
| Resting Membrane Potential (mV) | -70.77(1.24) | -69.73(1.69) | 0.62 |
|  |  |  |  |
| ***Trip8b-/-*** |  |  |  |
| Membrane Resistance (MΩ) | 81.8 (7.4) | 77.4 (7.4) | 0.70 |
| Resting Membrane Potential (mV) | -73.5 (1.17) | -72.2 (2.21) | 0.57 |
